# *GNB3* overexpression causes obesity and metabolic syndrome

**DOI:** 10.1371/journal.pone.0188763

**Published:** 2017-12-05

**Authors:** Alev Cagla Ozdemir, Grace M. Wynn, Aimee Vester, M. Neale Weitzmann, Gretchen N. Neigh, Shanthi Srinivasan, M. Katharine Rudd

**Affiliations:** 1 Department of Human Genetics, Emory University School of Medicine, Atlanta, GA, United States of America; 2 Department of Environmental Health Sciences, Rollins School of Public Health, Emory University, Atlanta, GA, United States of America; 3 Division of Endocrinology, Metabolism, and Lipids, Emory University School of Medicine, Atlanta, GA, United States of America; 4 Atlanta VA Medical Center, Decatur, GA, United States of America; 5 Department of Physiology, Emory University School of Medicine, Atlanta, GA, United States of America; 6 Department of Anatomy & Neurobiology, Virginia Commonwealth University, Richmond, VA, United States of America; 7 Division of Digestive Diseases, Emory University School of Medicine, Atlanta, GA, United States of America; University of Hong Kong, HONG KONG

## Abstract

The G-protein beta subunit 3 (*GNB3*) gene has been implicated in obesity risk; however, the molecular mechanism of *GNB3*-related disease is unknown. *GNB3* duplication is responsible for a syndromic form of childhood obesity, and an activating DNA sequence variant (C825T) in *GNB3* is also associated with obesity. To test the hypothesis that *GNB3* overexpression causes obesity, we created bacterial artificial chromosome (BAC) transgenic mice that carry an extra copy of the human *GNB3* risk allele. Here we show that *GNB3*-T/+ mice have increased adiposity, but not greater food intake or a defect in satiety. *GNB3*-T/+ mice have elevated fasting plasma glucose, insulin, and C-peptide, as well as glucose intolerance, indicating type 2 diabetes. Fasting plasma leptin, triglycerides, cholesterol and phospholipids are elevated, suggesting metabolic syndrome. Based on a battery of behavioral tests, *GNB3*-T/+ mice did not exhibit anxiety- or depressive-like phenotypes. *GNB3*-T/+ and wild-type animals have similar activity levels and heat production; however, *GNB3*-T/+ mice exhibit dysregulation of acute thermogenesis. Finally, *Ucp1* expression is significantly lower in white adipose tissue (WAT) in *GNB3*-T/+ mice, suggestive of WAT remodeling that could lead to impaired cellular thermogenesis. Taken together, our study provides the first functional link between *GNB3* and obesity, and presents insight into novel pathways that could be applied to combat obesity and type 2 diabetes.

## Introduction

Obesity is a chronic disease associated with significant morbidity and mortality, affecting over 600 million adults globally [1]. Furthermore, obesity is an important risk factor for metabolic conditions such as type 2 diabetes mellitus, insulin resistance, dyslipidemia, and cardiovascular problems including hypertension, cardiovascular disease, and congestive heart failure, as well as certain cancers [2–4]. Obesity is a highly heritable trait–twin and adoption studies estimate that over 70% of the variance in BMI is attributed to genetic factors [5,6]. Since the identification of leptin (*LEP*) as the first obesity gene [7], several other Mendelian forms of non-syndromic obesity have been discovered [8–10]. Along with monogenic forms of obesity, genetic disorders like Prader-Willi and Bardet-Biedl syndromes include obesity as a significant phenotype [9]. Moreover, genome-wide association studies have identified alleles that contribute to common forms of obesity [11]. Though many genetic causes have been discovered, additional genes are necessary to explain the “missing heritability” in obesity [12,13].

We recently described a syndrome associated with obesity, seizures, and intellectual disability in individuals with an unbalanced chromosome translocation that leads to an 8.5-megabase (Mb) duplication of chromosome 12 and a 7.0-Mb deletion of chromosome 8 [14]. One of the duplicated genes on chromosome 12 is the obesity candidate gene *GNB3*, which encodes the G-protein β3 subunit. Specific *in vivo* interactions of the Gβ subunits with other Gα and Gγ subunits are unknown [15,16]. However, a silent cytosine to thymine (C825T) polymorphism in *GNB3* is associated with hypertension and obesity [17]. This variant, located in exon 10 of *GNB3*, does not alter the amino acid sequence; however, the T-allele is associated with alternative splicing of exon 9 and encodes a splice variant (*GNB3*-s) with a 123-bp in-frame deletion [18]. The T-allele is also associated with increased signal transduction by activation of G-proteins in human cells [18]. *GNB3*-s produces a functional protein; yet, the properties of *GNB3*-s that enhance activation of G-proteins in unknown. Since Gβ subunits are important mediators of transmembrane signaling [19], *GNB3*-s could enhance signal transduction in a variety of tissues. How the *GNB3*-T allele, the associated splice variant, and increased G-protein signal transduction contribute to obesity risk are not understood.

Though *Gnb3* knock-out does not alter body weight in mice [20], our human data suggested that *GNB3* duplication leads to obesity. To model *GNB3* overexpression, we created transgenic mice carrying the T variant of human *GNB3* [14]. In addition to the two endogenous copies of *Gnb3*, *GNB3*-T/+ mice carry two copies of human *GNB3*-T. Previous work from our group demonstrated that heterozygous *GNB3* transgenic mice weigh significantly more than sex- and age-matched wild-type (WT) littermates and that *GNB3*-T is highly expressed in the brain [14]. Here, we build upon these findings and establish that *GNB3* overexpression causes increased adiposity, glucose intolerance, metabolic syndrome and dysregulation of acute thermogenesis in mice even though food intake, satiety, activity levels, energy expenditure, and behavioral phenotypes are similar to that of WT animals.

## Materials and methods

### Animals

Mice were housed in a 22–23°C climate controlled room on a 12-h light/dark cycle (lights on at 0700 h), in static micro-isolator cages with free access to water and standard rodent chow (Purina LabDiet 5001). Mice rooms were in an Association for Assessment and Accreditation of Laboratory Animal Care (AAALAC)-accredited facility in accordance with the National Research Council’s Guide for the Care and Use of Laboratory Animals. All animal studies were performed according to protocols approved by the Institutional Animal Care and Use Committee at Emory University. The BAC transgenic *GNB3*-T/+ mice were developed on a FVB background as previously described [14]. Mice were weighed once a week or once every 5 weeks from weaning to 25 weeks of age. Mice were euthanized by isoflurane.

### Food intake

Littermate mice were housed in groups of 2–3, separated by sex and genotype. Mice had free access to water and chow in cages that were supplied with a pre-weighed amount of food. For three days, the remaining food in the cage was weighed every 12 hours at ages 5, 10, 15, 20 and 25 weeks. Mice were acclimated to the cages for at least 24 hours before measuring food intake. Per mouse food consumption was calculated by dividing the total amount of food consumed in cage (g) by the number of animals in cage.

### Tissue weights

Inguinal and gonadal WAT, brown adipose tissue (BAT) and liver were dissected and weighed. Percent tissue weights were calculated by dividing tissue weight by total body weight of each mouse. Mouse length was measured from nose to anus.

### Dual-energy X-ray absorptiometry (DXA)

Body composition was measured using DXA scanning (Lunar PIXImus2 densitometer, GE Medical Systems) after anesthesia using isoflurane at ages 5 and 20 weeks.

### Blood analysis

Five and 20-week-old mice were fasted for 6 hours. Blood glucose was measured by a glucometer (Accu-Check Aviva, Roche) from a drop of tail blood collected by milking the tail. For metabolic, lipid and hormone profiling, blood was collected by cardiac puncture following euthanasia using isoflurane. Blood collection started between 1:00 and 2:00 pm each day. Dipeptidyl peptidase-4 inhibitor (EMD Millipore Corporation, Billerica, MA, USA), aprotinin (Sigma-Aldrich, Saint Louis, MO, USA), protease inhibitor cocktail (Sigma-Aldrich, Saint Louis, MO, USA) and serine protease inhibitor (AEBSF) (Sigma-Aldrich, Saint Louis, MO, USA) were added to samples collected in tubes coated with EDTA (Becton, Dickinson and Company, Franklin Lakes, NJ). Fasting blood plasma was separated immediately by centrifuge (1000×g) for 10 minutes at room temperature and was aliquoted and stored at −20°C. Plasma concentrations of amylin (active), C-peptide, acylated ghrelin (active), GIP (total), GLP-1 (active), glucagon, IL-6, insulin, leptin, MCP-1, PP, PYY, resistin and TNF-α were measured using MILLIPLEX MAP mouse metabolic hormone magnetic bead panel kit (MMHMAG-44K). Plasma concentrations of ACTH, FSH, GH, prolactin, TSH and LH were measured using MILLIPLEX MAP mouse pituitary magnetic bead panel kit (MPTMAG-49K) (Millipore, Billerica, MA, USA). The assays were performed according to the manufacturer’s instructions. Plasma triglycerides, cholesterol, phospholipids and non-esterified fatty acids were measured at the Mouse Metabolic Phenotyping Center (Cincinnati, OH, USA).

### Glucose tolerance test and insulin tolerance test

For the glucose tolerance test (GTT), 5 and 20-week-old mice were fasted for 6 hours and injected intraperitoneally with 1 mg/g D-glucose (Sigma-Aldrich, St. Louis, MO, USA). For the insulin tolerance test (ITT), 20-week-old mice were fasted for 4 hours and injected intraperitoneally with insulin (I9278 SIGMA, Sigma-Aldrich, St. Louis, MO, USA) using 0.05 units/kg (females) or 0.1 units/kg (males). Blood glucose levels were measured from the tip of the tail using a glucometer (Accu-Check Aviva, Roche) at -5, 0, 5, 10, 15, 20, 25, 30, 60, 120 min after injection for GTT, and at 0, 15, 30, 45, 60, 75, 90 min after injection for ITT, respectively.

### Indirect calorimetry study

Five and 20–22 week old male mice were housed individually in metabolic chambers with free access to food and water on a 12 hour light/dark cycle, and assessed for metabolic activities using an OPTO-M3 sensor system (Oxymax, Columbus Instruments, Columbus, OH, USA). Spontaneous activity, volume of oxygen consumption (VO_2_) and volume of carbon dioxide production were measured over a 72 hour collection period after 48 hours of acclimation.

### Cold challenge: Rectal temperature measurement

Rectal temperature of 5 and 20 week old mice was measured using a MicroTherma 2T hand held thermometer (ThermoWorks, Lindon, UT, USA) at time 0, 15, 30, 45, 60, 75 min after placement in a 4°C room.

### Behavioral tests

We used an established repertoire of tests [21] to examine anxiety- and depressive-like behaviors in adult *GNB3*-T/+ and WT mice. Mice were between 19–23 weeks old and behavioral tests were started between 2:00 and 3:00 pm each day. The same investigator conducted all experiments and was blinded to treatment group. Both groups were counterbalanced. Behavioral tests were conducted on subsequent days in the following order: open field testing and novelty suppressed feeding, social interaction, marble burying, novel object recognition. For all behavioral assays, 6 *GNB3*-T/+ and 6 WT subjects were used.

The open field test measures both general locomotor activity and anxiety-like behavior [22–24]. Mice were placed in a corner of a 45x45 cm^2^ square box and allowed to explore for 10 minutes. Noldus Ethovision software was used to record and analyze total distance moved, frequency of entrance into the center zone, time spent in center zone, and latency to enter center zone.

Novelty suppressed feeding measures the latency to feed in a novel environment and is sensitive to administration of anti-depressants and anxiolytics [25]. Mice were tested on the same day as the open field test to ensure animals were not habituated to the environment. Three grams of sucrose pellets were placed in the center of the open field box and mice were allowed to freely explore for 10 minutes. After testing, mice were placed back in the home cage along with the sucrose pellets and were allowed to feed for an additional 5 minutes. The sucrose pellets left over were weighed and subtracted from the initial amount to determine total amount of sucrose eaten. All mice were habituated to sucrose pellets at least 24 hours before testing.

Social interaction is also a measure of anxiety-like behavior in mice [26,27] and was assessed with age-matched, non-littermate mice as a stimulus. The stimulus mouse was placed at the center of the open field box and the reactive mouse was placed in a corner of the open field box. Mice were allowed to interact for 10 minutes. Latency to interact and total interaction time were analyzed.

The marble burying test also examines anxiety-like behavior [28]. Using clean mouse cages with twice the normal amount of bedding, twenty marbles were placed on top of bedding in a 4x5 pattern. Mice were placed in a corner of the box and allowed to explore for 30 minutes. After testing mice were removed and two researchers independently counted the number of marbles buried.

The novel object recognition task is used to assess learning and memory in mice [29,30]. Testing was conducted in the open field box over three successive days. For each round of testing, mice were placed in the same corner of the open field box. Two identical objects were placed in opposite corners of the box and mice were allowed to explore freely for 10 minutes. Mice were habituated to the test set-up for one day before testing. For the no-delay test, one of the objects was replaced by a novel object immediately after. For the one-hour and 24-hour delay tests, mice were placed back in the home cage for one or 24 hours, respectively. Mice were then placed back in the box with a different novel object and allowed to explore for 10 minutes. Noldus Ethovision software was used to record and analyze number of object touches, time spent sniffing each object, latency to approach objects, total distance moved, and average velocity.

### Histology

Inguinal and gonadal WAT were fixed in 10% formalin. Tissue processing, embedding, sectioning and haematoxylin and eosin (H&E) staining were performed at Emory University Winship Pathology Core Lab. Adipocyte size was measured using ImageJ software [31].

### Gene expression: Total RNA extraction, cDNA synthesis, and quantitative real-time PCR

Total RNA was extracted from fresh tissue using the RNeasy Lipid Tissue Mini kit (Qiagen, Austin, TX, USA) following manufacturer’s instructions and cDNA was synthesized from total RNA using the SuperScript III First-Strand Synthesis System for RT-PCR (Invitrogen Corporation, Carlsbad, CA, USA). Quantitative real-time PCR was performed using iQ SYBR Green Supermix (Bio-Rad Laboratories, Hercules, CA, USA) mixed with gene-specific primers on the Bio-Rad CFX96 Real-Time PCR Detection System. Expression data were normalized by the 2-[delta][delta]Ct method using *Gapdh* as an internal control. Primer sequences are listed in S1 Table. Taqman quantitative RT-PCR was performed as described [14] to measure *GNB3* and *Gnb3* expression using *Actb* as an internal control.

### Protein extraction and western blot analysis

Inguinal WAT (iWAT) and BAT were dissected, weighed and immediately homogenized on ice in CelLytic MT Mammalian Tissue Lysis/Extraction Reagent (Sigma-Aldrich, St. Louis, MO, USA) with protease inhibitor cocktail. Total protein concentration was determined using Pierce BCA Protein Assay Kit (Pierce Biotechnology, Rockford, IL, USA).

### Citrate synthase

Citrate synthase activity for iWAT and BAT extracts were measured using the Citrate Synthase Assay Kit (Sigma-Aldrich, St. Louis, MO, USA) following the manufacturer’s instructions.

### Statistical analysis

Statistical analyses were performed using GraphPad Prism version 6 for Mac (GraphPad Software, Inc., La Jolla, CA, USA). Data are presented as mean ± SD (or SEM where indicated). Unpaired Student’s *t*-test was used to compare two groups, and one-way ANOVA was used to compare more than two groups. Comparisons with *p*-values <0.05 were considered significant.

## Results

### Transgenic *GNB3* expression in brain and adipose tissue

**Fig 1:**
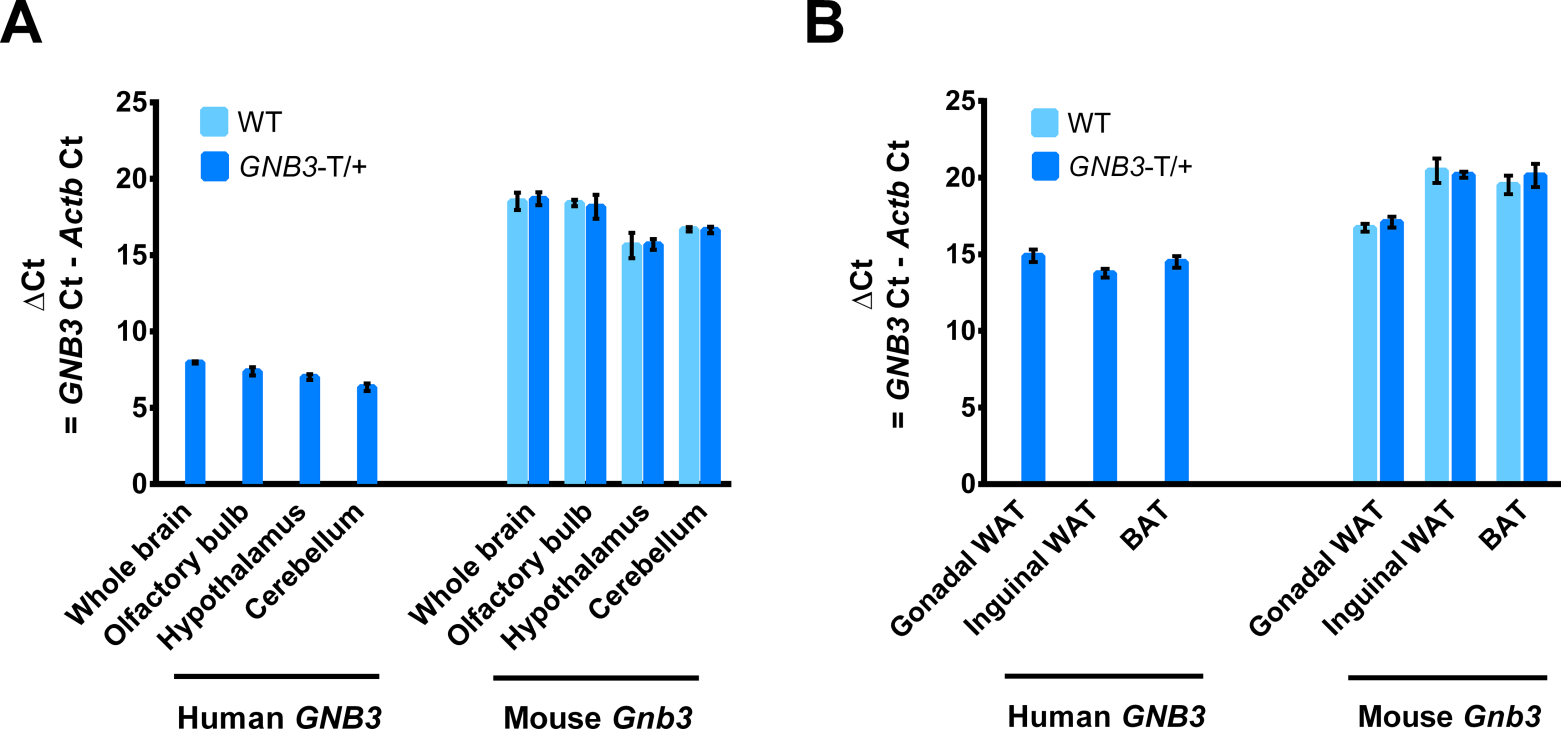
Transgenic *GNB3* is highly expressed in the brain, at levels greater than endogenous *Gnb3*. A. Transgenic human *GNB3* and mouse endogenous *Gnb3* gene expression in brain tissues of 5-week-old mice. B. *GNB3* and *Gnb3* expression in gWAT, iWAT, and BAT of 20-week-old mice. *n* = 4 mice per group in A, *n* = 3 mice per group in B. 3 technical replicates for both. Ct, cycle threshold.

In this study, we refer to mice heterozygous for the human BAC transgene as *GNB3*-T/+. To determine the expression levels of human *GNB3* and endogenous *Gnb3* we performed quantitative RT-PCR in RNA from whole brain, olfactory bulb, hypothalamus, and cerebellum of 5-week-old *GNB3*-T/+ and WT mice. As expected, we did not detect human *GNB3* in WT mice. Notably, human *GNB3* expression was much greater than endogenous *Gnb3* in whole brain, olfactory bulb, hypothalamus and cerebellum of *GNB3*-T/+ mice as calculated by delta cycle threshold values (Fig 1A). We also detected expression of human *GNB3* and endogenous *Gnb3* in adipose tissue from 20-week-old *GNB3*-T/+ and WT mice. Human *GNB3* expression was 4-fold greater than endogenous *Gnb3* in gonadal WAT (gWAT), and 50-fold greater than endogenous *Gnb3* in iWAT and BAT (Fig 1B).

### Greater adiposity in *GNB3*-T/+ mice

**Fig 2:**
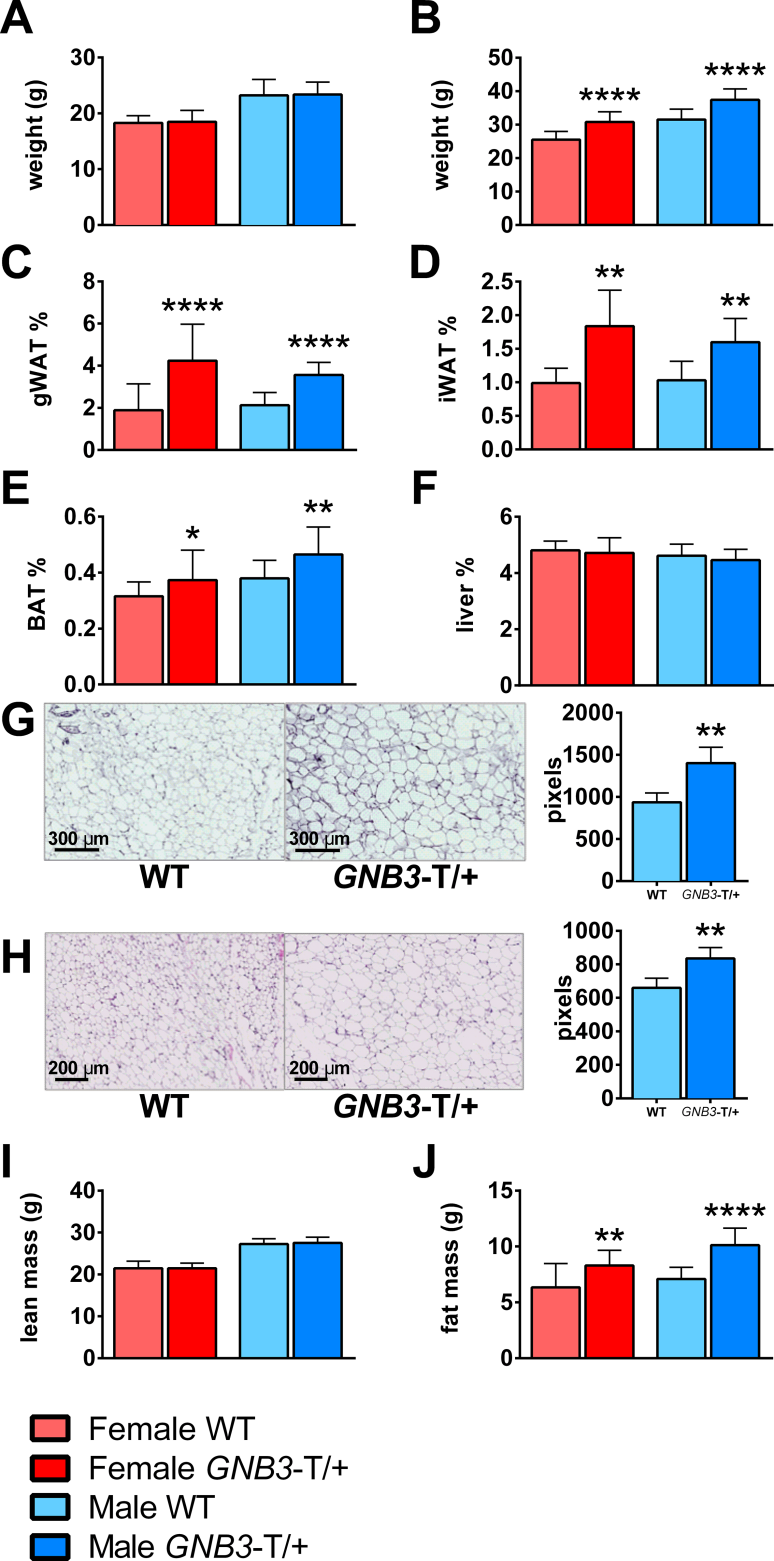
*GNB3*-T/+ mice have greater adiposity than WT. A. Body weight of 5-week-old mice. B. Body weight, C. gWAT weight/body weight (gWAT%), D. iWAT weight/body weight (iWAT%), E. BAT weight/body weight (BAT%), F. liver weight/body weight (liver%) of 20-week-old mice. G. Representative images of 20-week-old gWAT and H. iWAT sections stained with H&E; cell size measured in pixels to the right. I. DXA lean mass and J. DXA fat mass of 20-week-old mice. *n* = 8–11 mice per group in A; *n* = 16–23 mice per group in B, C and E; *n* = 5–9 mice per group in D; *n* = 10–20 mice per group in F; *n* = 5 mice per group in G, H (five images per mouse were used to quantify adipocyte size in pixels); *n* = 17–20 mice per group in I, J. Data are mean ± SD. *P < 0.05, **P < 0.01, ***P < 0.001, ****P < 0.0001 vs. WT of same sex by unpaired Student’s *t*-test.

In our previous studies, we found that *GNB3*-T/+ mice weighed significantly more than WT littermates starting at age 6–7 weeks onwards [14]. We chose two ages, 5 weeks and 20 weeks, to evaluate the phenotypes of mice before and after obesity onset (Fig 2A and 2B) and weighed gWAT and iWAT as representative of visceral and subcutaneous WAT depots, respectively (S2 Table). At 5 weeks, *GNB3*-T/+ and WT mice had similar gWAT%, iWAT%, BAT%, and liver% (S1A–S1D Fig). However, at 20 weeks, female and male *GNB3*-T/+ mice had greater gWAT%, iWAT%, and BAT%, but similar liver% compared to WT (Fig 2C–2F). H&E staining revealed *GNB3*-T/+ adipocytes were 50% larger in gWAT (Fig 2G) and 27% larger in iWAT (Fig 2H) depots compared to WT. To further investigate body composition, we performed DXA on *GNB3*-T/+ and WT mice. Though lean mass of *GNB3*-T/+ and WT mice was the same at 5 and 20 weeks (Fig 2I, S1 Fig), fat mass was increased in male and female *GNB3*-T/+ mice at 20 weeks (Fig 2*J*).

### *GNB3*-T/+ mice have metabolic syndrome

**Fig 3:**
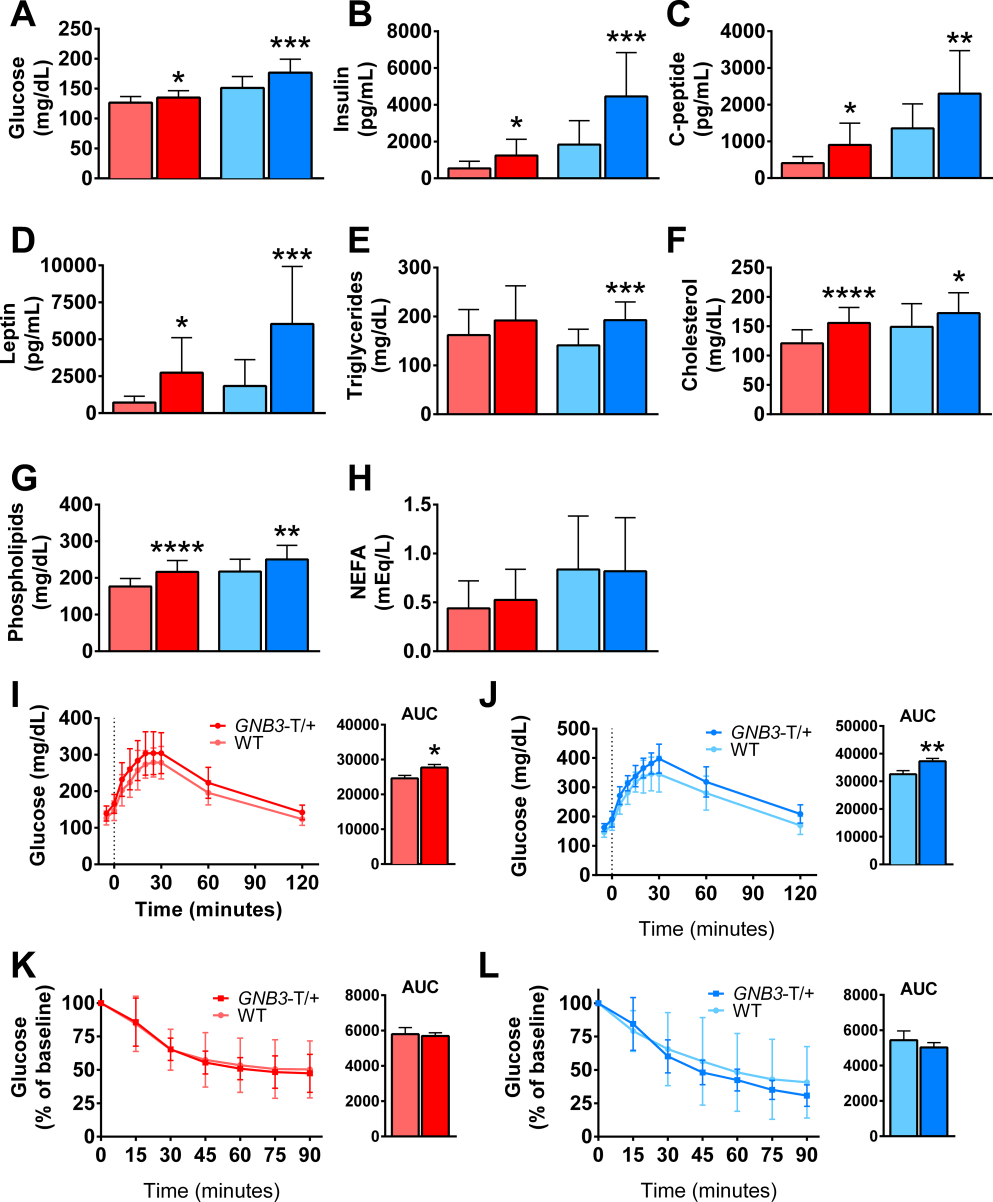
*GNB3*-T/+ mice have metabolic syndrome. A. Fasting blood glucose, B. fasting plasma insulin, C. C-peptide, D. leptin, E. triglycerides, F. cholesterol, G. phospholipids and H. non-esterified fatty acids of mice. I. GTT and areas under the curve (AUC) of female and J. male mice. K. ITT and AUC of female and L. male mice. All mice are 20 weeks old. *n* = 15–23 mice per group in A, *n* = 11–20 mice per group in B-D, *n* = 16–25 mice per group in E-H, *n* = 18–22 mice per group in I, *n* = 17–18 mice per group in J, *n* = 14–16 mice per group in K, *n* = 10–17 mice per group in L. Data are mean ± SD. *P < 0.05, **P < 0.01, ***P < 0.001, ****P < 0.0001 vs. WT of same sex by unpaired Student’s *t*-test. Colors are the same as in Fig 2.

Next, we investigated the metabolic profiles of *GNB3*-T/+ mice prior to and during obesity. At 20-weeks-old, female and male *GNB3*-T/+ mice had elevated fasting blood glucose, fasting plasma insulin, and C-peptide compared to WT (Fig 3). However, at 5 weeks, fasting blood glucose was greater only in female *GNB3*-T/+ mice, but female and male *GNB3*-T/+ mice had elevated fasting plasma insulin (S2A and S2B Fig). Lipid profiling revealed that at 5 weeks, fasting plasma leptin, triglycerides, and cholesterol were similar for *GNB3*-T/+ mice and WT in both sexes (S2D–S2F Fig). Phospholipids were elevated, and non-esterified fatty acids (NEFA) were lower in female *GNB3*-T/+ mice, while in males both were similar to WT (S2G and S2H Fig). At 20 weeks, *GNB3*-T/+ mice had higher fasting plasma leptin, triglycerides, cholesterol, and phospholipids; however, NEFA were similar for *GNB3*-T/+ mice and WT (Fig 3).

To evaluate the impact of *GNB3* overexpression on glucose metabolism, we subjected mice to a GTT prior to and during obesity. At 5 weeks of age, *GNB3*-T/+ and WT mice have similar glucose tolerance (S2I and S2J Fig). However, at 20 weeks, as indicated by glycemia levels and calculations of area under the curve (AUC), female and male *GNB3*-T/+ mice exhibited glucose intolerance (Fig 3I and 3J). To follow up these findings, we assessed insulin sensitivity at 20 weeks old via ITT. For both sexes, insulin sensitivity was similar between *GNB3*-T/+ and WT mice (Fig 3K and 3L), though fasting insulin was elevated in *GNB3*-T/+ mice (Fig 3B). This difference could be explained by the low insulin concentration used in the ITT, which was determined empirically.

Further metabolic profiling revealed that prior to obesity, fasting plasma glucagon, resistin and gastric inhibitory polypeptide (GIP) were not significantly different in *GNB3*-T/+ and WT mice (S3A, S3C and S3E Fig). At 20 weeks of age, resistin was elevated only in male *GNB3*-T/+ mice, while *GNB3*-T/+ glucagon and GIP levels were not different than WT in either sex (S3B, S3D and S3F Fig). Inflammatory marker IL-6, TNF-alpha and MCP-1 levels in fasting plasma were similar between *GNB3*-T/+ and WT mice prior to and during obesity (S3G–S3L Fig). Growth hormone (GH), thyroid stimulating hormone (TSH), follicle stimulating hormone (FSH), luteinizing hormone (LH), prolactin, and adrenocorticotropic hormone (ACTH) levels were similar between *GNB3*-T/+ and WT mice at 5 weeks. However, at 20 weeks, GH, TSH, FSH, LH and prolactin were lower in obese *GNB3*-T/+ male mice (S4 Fig). It is important to note that GH secretion is pulsatile, so we measured GH at the same time point each day to minimize variation.

### No significant difference detected between *GNB3*-T/+ and WT mice in food intake and activity levels

**Fig 4:**
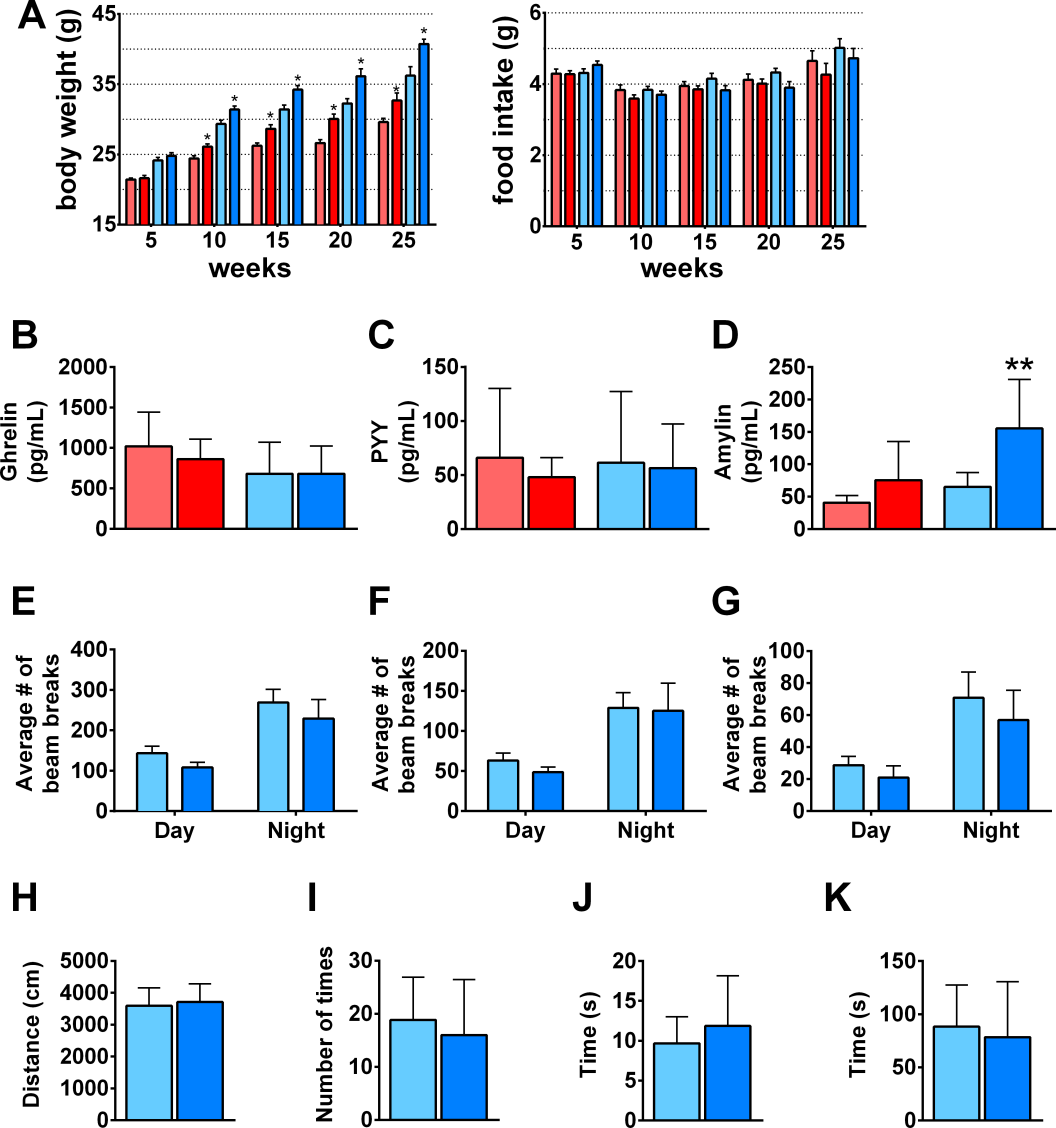
*GNB3*-T/+ mice have proper satiety, similar food intake and activity levels compared to WT. A. Daily amount of food consumption per mouse (right) and corresponding body weight of same mice (left) measured every 5 weeks for 25 weeks. B. Fasting plasma ghrelin, C. PYY, D. amylin. E. Total movement in X-plane, F. horizontal ambulatory activity, and G. vertical activity averaged over a 72-hour period. H. Distance moved, I. frequency of entrance into center zone, J. time spent in center, K. latency during open field test. Mice are 20 weeks old in B-K. *n* = 24–33 mice per group in A, *n* = 10–13 mice per group in B, *n* = 7–19 mice per group in C, *n* = 4–13 mice per group in D, *n* = 16–31 mice per group in E-G, *n* = 6–7 mice per group in H-K. Data are mean ± SD in B-D, H-K; and mean ± SEM in A, E-G. *P < 0.05, **P < 0.01 vs. WT of same sex by unpaired Student’s *t*-test. Colors are the same as in Fig 2.

The increased adiposity and total body weight in *GNB3*-T/+ mice could be due to greater food intake, lack of activity, or a metabolic defect in energy expenditure. To investigate these possibilities, we measured food consumption at ages 5, 10, 15, 20 and 25 weeks (Fig 4A). At each time point, food intake was not significantly different between *GNB3*-T/+ and WT mice. Prior to obesity, fasting plasma ghrelin was lower, but PYY and amylin were elevated in male *GNB3*-T/+ mice; no difference was detected between *GNB3*-T/+ and WT females (S5A–S5C Fig). At 20 weeks of age, *GNB3*-T/+ fasting plasma ghrelin and PYY levels were similar to WT, while amylin was elevated in *GNB3*-T/+ mice (Fig 4), suggesting proper satiety.

Next, we measured locomotor activity in *GNB3*-T/+ mice over the course of three days. Horizontal and vertical activity levels during day and night cycles were not significantly different between *GNB3*-T/+ and WT mice at 5 weeks (S5D–S5F Fig) or 20 weeks of age (Fig 4E–4G). In addition, there was no significant difference in total distance moved, frequency of center zone entrances, time spent in the center zone, or latency to center zone between the *GNB3*-T/+ and WT mice in open field testing (Fig 4H–4K). This suggests that there is not a general locomotor defect in *GNB3*-T/+ mice, or an anxiety-like phenotype that would affect locomotor activity.

### Energy expenditure and *Ucp1* expression in *GNB3*-T/+ mice

**Fig 5:**
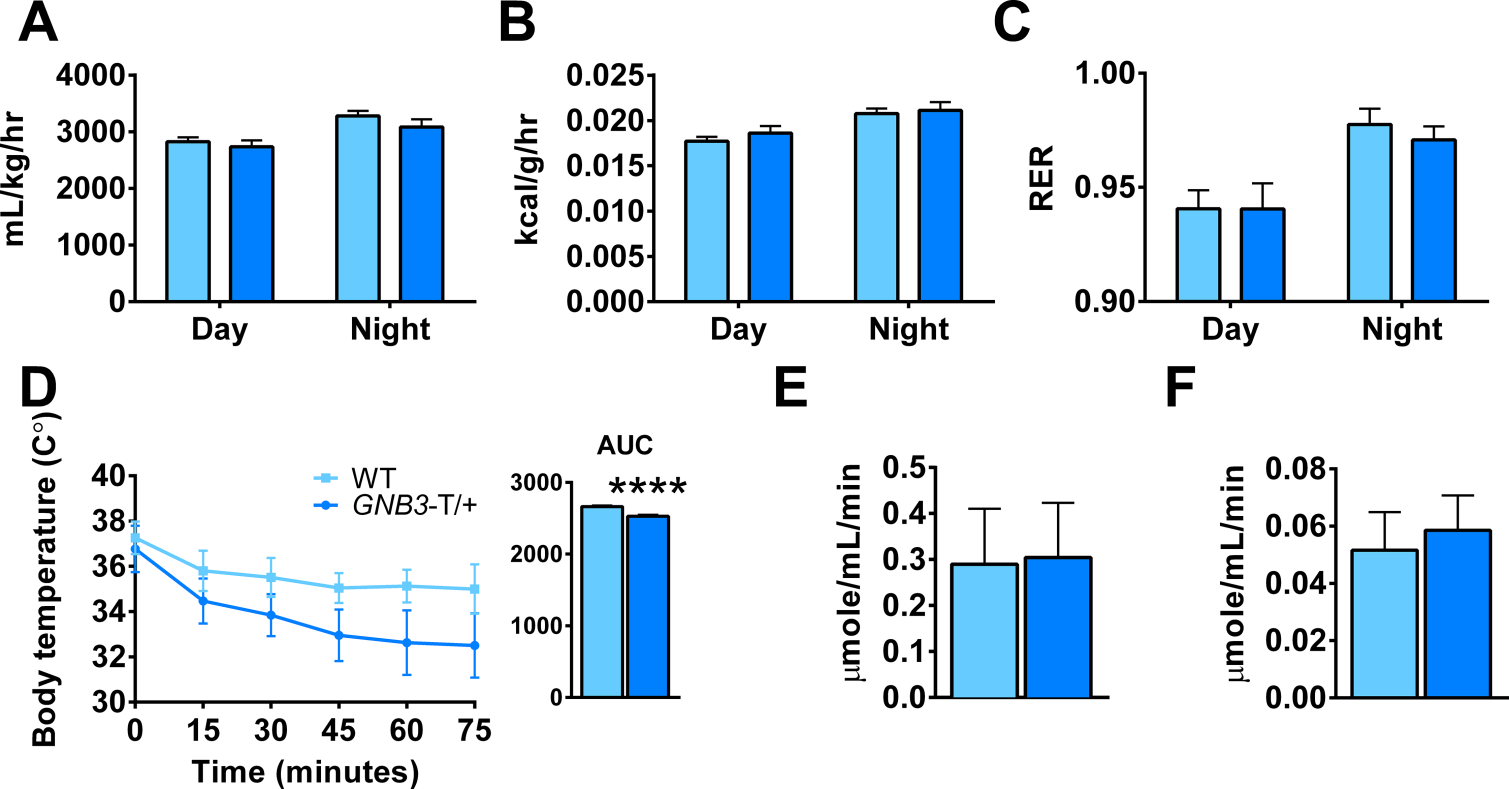
Oxygen consumption is similar in *GNB3*-T/+ and WT mice, but *GNB3*-T/+ mice have dysregulation of acute thermogenesis. A. VO_2_, B. heat produced normalized over body weight, and C. RER (VCO_2_/VO_2_) averaged over a 72-hour period. D. Acute cold stress at 4°C, E. citrate synthase activity in BAT and F. iWAT. All mice are 20 weeks old. *n* = 16–31 mice per group in A-C, *n* = 8–14 mice per group in D, *n* = 13–14 mice per group in E, F. Data are mean ± SEM in A-C; and mean ± SD in D-F. ****P < 0.0001 vs. WT of same sex by unpaired Student’s *t*-test.

Since food intake and locomotor activity were similar between *GNB3*-T/+ and WT mice, we next considered energy expenditure. Using metabolic cages, we measured VO_2_, heat production, and respiratory exchange ratio (RER) in male mice. At 5 weeks old, VO_2_, heat and RER measurements are similar in *GNB3*-T/+ and WT mice (S6A–S6C Fig). Once obese, *GNB3*-T/+ mice consume less oxygen, though the difference is not statistically significant. Heat production and RER in *GNB3*-T/+ mice are comparable to WT (Fig 5).

To further probe differences in energy expenditure, we performed an acute cold challenge at 4°C for 75 minutes and monitored the core body temperature of mice. Prior to obesity, *GNB3*-T/+ and WT mice dropped their core body temperatures at a similar rate during acute cold stress (S6D Fig). However, at 20 weeks old, *GNB3*-T/+ mice had significantly lower core body temperature compared to WT (Fig 5D). Since failure to maintain body temperature during acute cold exposure could be related to BAT function, we measured citrate synthase, a marker of mitochondrial activity [32]. Citrate synthase activity was comparable in BAT and iWAT from 20-week-old *GNB3*-T/+ and WT mice (Fig 5E and 5F).

### Gene expression in *GNB3*-T/+ mice

**Fig 6:**
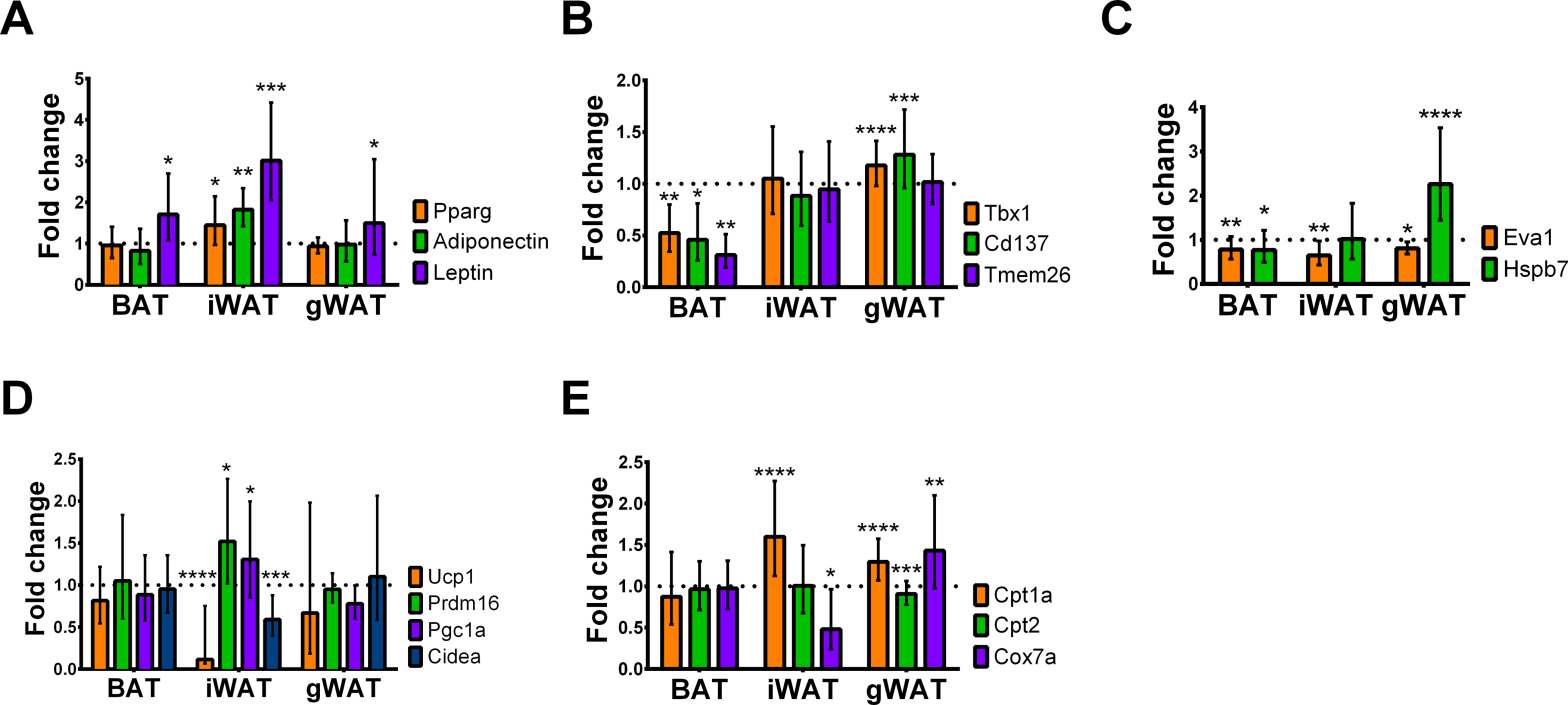
*GNB3*-T/+ mice have lower gene expression of *Ucp1* in BAT, iWAT and gWAT, and lower gene expression of beige and brown adipocyte markers in BAT. A. Gene expression profiles of adipogenic, B. beige adipocyte, C. brown adipocyte, D. oxidative phosphorylation, and E. mitochondria markers in BAT, iWAT and gWAT measured by quantitative RT-PCR. Fold change is *GNB3*-T/+ versus WT, mean ± SD. All mice are 20 weeks old. *n* = 3 mice per group and experiment was repeated 3 times. *P < 0.05, **P < 0.01, ***P < 0.001, ****P < 0.0001 vs. WT of same sex by unpaired Student’s *t*-test.

We measured expression of oxidative phosphorylation, mitochondria, and white, beige, and brown adipocyte markers by quantitative RT-PCR in BAT, iWAT, and gWAT, and calculated the fold change between *GNB3*-T/+ and WT mice. Leptin expression was increased in BAT, iWAT and gWAT. Adipogenic markers *Pparg* and adiponectin were elevated in iWAT (Fig 6A). Beige adipocyte markers *Tbx1*, *Cd137*, and *Tmem26* (Fig 6B) as well as brown adipocyte markers *Eva1* and *Hspb7* had lower expression in BAT. *Eva1* expression was also lower in iWAT and gWAT, while *Hspb7* expression was elevated in gWAT (Fig 6C). Expression of *Ucp1*, a marker of brown and beige adipocytes, was lower most dramatically in iWAT. Additionally, iWAT *Prdm16* and *Pgc1a* expression were elevated, while *Cidea* expression was lower (Fig 6D). Mitochondrial genes *Cpt1a*, *Cpt2* and *Cox7a* were equally expressed in BAT. *Cpt1a* was elevated in iWAT and gWAT, while *Cox7a* was lower in iWAT but elevated in gWAT (Fig 6E).

## Discussion

The mechanism of *GNB3*-related disease is only beginning to be understood, yet G-proteins are excellent candidates for a role in obesity [33]. By transducing extracellular signals, membrane-spanning G-protein coupled receptors activate G-protein αβγ heterotrimers [34]. Activated Gα, Gβ, and Gγ subunits are critical molecules that transmit signals to intracellular signaling pathways [35]. G-protein mediated signaling, by combinations of different Gα, Gβ, and Gγ subunit isoforms, controls diverse cellular and organismal functions such as differentiation, sensation, growth, and homeostasis [33]. Mutations in some G-protein subunit genes lead to abnormal signal transduction [36,37]. For example, impaired taste sensation, defects in metabolism, and a variety of endocrine disorders are caused by mutations in different human Gα subunits [33,37,38].

In our transgenic model, *GNB3* overexpression is associated with obesity, type 2 diabetes, and metabolic syndrome that presents without hyperphagia or reduced locomotion. Specifically, we find fat accumulation in visceral and subcutaneous WAT depots as well as in BAT. The lean mass of *GNB3*-T/+ and WT mice is the same, indicating that the difference in weight is strictly due to fat mass. Even though livers of *GNB3*-T/+ mice weighed more than WT, when normalized to total body weight (liver %), this difference was not significant.

Glucose intolerance and type 2 diabetes are apparent at 20 weeks in *GNB3*-T/+ mice, indicated by elevated fasting plasma glucose levels and GTT response. Though *GNB3*-T/+ mice did not have glucose intolerance prior to obesity, 5-week old female *GNB3*-T/+ mice had elevated fasting blood glucose, and both female and male *GNB3*-T/+ mice had elevated fasting plasma insulin. This could indicate the beginning stage of impaired glucose metabolism prior to obesity. However, *GNB3*-T/+ mice do not respond to the ITT like other mouse models of type 2 diabetes, indicating a milder phenotype.

GH was reduced in obese *GNB3*-T/+ mice, consistent with lower circulating GH in obese humans [39]. Another pituitary hormone, TSH, was also reduced in obese *GNB3*-T/+ mice at 20 weeks. Thyroid hormones control multiple physiological systems and have an important role in regulating basal metabolic rate, lipolysis, as well as the differentiation process in the adipose tissue [40]. FSH, LH and prolactin are reduced in obese male *GNB3*-T/+ mice at 20 weeks, which could indicate hypogonadotropic hypogonadism [41].

Obesity is caused by an energy imbalance between calories consumed and calories expended. The increased adiposity in *GNB3*-T/+ mice could be due to increased calorie intake, reduced activity, or a defect in metabolism that results in lower energy expenditure. Our results from food intake measurements and levels of fasting ghrelin, PYY, and amylin hormones revealed that hyperphagia or a satiety defect are not responsible. Novelty suppressed feeding tests also show no significant difference in the amount of sucrose eaten, latency to feed, or total feeding time between the *GNB3*-T/+ and WT mice, indicating that anxiety-like feeding behaviors are not involved in *GNB3*-related obesity. *GNB3*-T/+ and WT mice do not have a statistically significant difference in locomotor activity or oxygen consumption. However, it is possible that subtle differences in locomotion and/or oxygen consumption could contribute to increased adiposity. Future energy expenditure experiments conducted at thermoneutrality and/or brown fat induction experiments could shed light on the effects of *GNB3* overexpression. Further, behavioral assessments indicate that there are no substantial anxiety- or depressive-like phenotypes in *GNB3*-T/+ mice, and that these affective phenotypes are unlikely to add to the relationship between *GNB3* overexpression and obesity.

Ucp1 expression in adipose tissues provides a clue to the underlying defect in *GNB3*-T/+ mice. Ucp1 in mitochondria dissipates chemical energy in the form of heat, mainly in BAT, through a process called nonshivering thermogenesis [42]. Recently, BAT has become a therapeutic target in obesity and metabolic disorders. Ucp1-ablated mice are obese and have type 2 diabetes, though this only occurs when living at thermoneutrality [43]. In addition to classic brown fat, white fat depots contain UCP1^+^ cells [44–48] known as beige [49] or brown-white (brite) adipocytes [50]. Beige cells and classic brown adipocytes have distinctly different molecular signatures [49] and developmental origins [51]. Adult humans have UCP1^+^ adipose tissue which has a gene expression pattern more similar to beige cells than to classic brown cells in the mouse [49]. Though *GNB3* overexpression alters the gene expression profiles of both BAT and WAT, *GNB3* appears to have the greatest effect in beige-cell containing iWAT.

BAT in *GNB3*-T/+ mice has lower expression of beige and brown adipocyte markers. Additionally, *GNB3*-T/+ mice showed markedly worsened beige adipocyte function in subcutaneous fat pads as indicated by lower levels of *Ucp1* in iWAT. Subcutaneous adipose tissue in *GNB3*-T/+ mice acquired properties of visceral fat indicated by elevated expression of adipogenic markers, *Pparg* and adiponectin. Overall, *GNB3* overexpression stimulates a conversion of subcutaneous WAT, particularly in the inguinal depot, into a less UCP1^+^ and a less beige but whiter tissue. We show that this white-like remodeling of iWAT and loss of brown and beige properties in BAT is accompanied by increased adiposity in mice fed normal chow. Together, these data implicate *GNB3* overexpression in impaired WAT and BAT, and for the first time provide a functional link between *GNB3* and obesity pathogenesis. However, the specific causes of *GNB3*-related obesity remain to be determined. Future studies of *GNB3* overexpression are needed to dissect the molecular mechanisms by which *GNB3* alters adipose tissue metabolism, signaling, and energy expenditure.

## Supporting information

S1 Fig Five-week-old *GNB3*-T/+ mice have similar adiposity compared to WT. A. gWAT weight/body weight (gWAT%), B. iWAT weight/body weight (iWAT%), C. BAT weight/body weight (BAT%), D. liver weight/body weight (liver%), E. DXA lean mass and F. DXA fat mass of 5-week-old mice. *n* = 8–11 mice per group in A, C, and D; *n* = 1–8 mice per group in B; *n* = 14–22 mice per group in E, F. Data are ± SD. No significant difference between *GNB3*-T/+ and WT of same sex by unpaired Student’s *t*-test.
(TIF)

S2 Fig *GNB3*-T/+ mice have slightly impaired glucose metabolism prior to obesity. A. Fasting blood glucose, B. fasting plasma insulin, C. C-peptide, D. leptin, E. triglycerides, F. cholesterol, G. phospholipids and H. non-esterified fatty acids. I. GTT and AUC of female and J. male mice. All mice are 5 weeks old. *n* = 8–11 mice per group in A, *n* = 7 mice per group in B-D, *n* = 9–13 mice per group in E-H, *n* = 10–14 mice per group in I, *n* = 11–15 mice per group in J. Data are ± SD. *P < 0.05, **P < 0.01 vs. WT of same sex by unpaired Student’s *t*-test.
(TIF)

S3 Fig Panel of metabolic hormones and inflammatory markers. **Male *GNB3*-T/+ mice have elevated fasting plasma resistin during obesity.** A,B. Fasting plasma glucagon; C,D. resistin; E,F. GIP; G,H. IL-6; I.J., TNF-a; and K,L. MCP-1 in 5-week and 20-week-old mice, respectively. *n* = 7 mice per group in A, C, E, G, I, K; *n* = 10–13 mice per group in B, *n* = 11–20 mice per group in D, F, H, J, L. Data are ± SD. *P < 0.05 vs. WT of same sex by unpaired Student’s *t*-test.
(TIF)

S4 Fig Panel of pituitary hormones. ***GNB3*-T/+ male mice have lower GH, TSH, FSH, LH and prolactin during obesity.** A,B. Fasting plasma growth hormone (GH); C,D. thyroid-stimulating hormone (TSH); E,F. follicle-stimulating hormone (FSH); G,H. luteinizing hormone (LH); I,J. prolactin; K,L. adrenocorticotropic hormone (ACTH) levels of 5 week and 20 week old mice, respectively. *n* = 7 mice per group in A, C, E, G, I, K; *n* = 11–20 mice per group in B, D, F, H, J, L. Data are ± SD. *P < 0.05, **P < 0.01, ***P < 0.001 vs. WT of same sex by unpaired Student’s *t*-test.
(TIF)

S5 Fig *GNB3*-T/+ mice have proper satiety and similar activity levels compared to WT prior to obesity. A. Fasting plasma ghrelin, B. PYY and C. amylin. D. Total movement in X-plane, E. horizontal ambulatory activity, and F. vertical activity averaged over a 72-hour period. All mice are 5 weeks old. *n* = 7 mice per group in A-C, *n* = 19–21 mice per group in D-F. Data are ± SD in A-C, and ± SEM in D-F. *P < 0.05, **P < 0.01, ***P < 0.001 vs. WT of same sex by unpaired Student’s *t*-test.
(TIF)

S6 Fig Oxygen consumption is similar in *GNB3*-T/+ and WT mice, and *GNB3*-T/+ mice do not have dysregulation of acute thermogenesis prior to obesity. A. VO_2_, B. heat produced normalized over body weight, and C. RER (VCO_2_/VO_2_) averaged over a 72-hour period. D. Acute cold stress at 4°C. All mice are 5 weeks old. *n* = 19–21 mice per group in A-C, *n* = 5–8 mice per group in D. Data are ± SEM in A-C; and ± SD in D-F. ****P < 0.0001 vs. WT of same sex by unpaired Student’s *t*-test.
(TIF)

S1 Table Sequence of primers used for real-time quantitative reverse transcription PCR. (DOCX)

S2 Table Absolute weights (g) of dissected adipose tissues and liver. (DOCX)

S3 Table Summary of behavioral assessment of GNB3-T/+ mice. GNB3-T/+ mice and WT littermates were subjected to behavioral tests in order to evaluate anxiety/depressive-like behaviors and learning and memory.
(DOCX)
