# Supplementary material for: GNB3 overexpression causes obesity and metabolic syndrome
Source: PLoS One. 2017 Dec 5;12(12):e0188763. doi: 10.1371/journal.pone.0188763 (PMC5716578; doi:10.1371/journal.pone.0188763)
Supplement: S1 Table — (DOCX) [file pone.0188763.s007.docx]

**S1 Table. Sequence of primers used for real-time quantitative reverse transcription PCR.**

| **Gene** | **Forward primer (5’🡪3’)** | **Reverse primer (5’🡪3’)** |
| --- | --- | --- |
| *Adipoq* | GGAACTTGTGCAGGTTGGAT | CCTTCAGCTCCTGTCATTCC |
| *Cd137* (or *Tnfrsf9*) | ATAGGTGGACAGCCGAACTG | GCCTGCAGTCCTTTTCACAT |
| *Cidea* | TGACATTCATGGGATTGCAG | TAACCAGGCCAGTTGTGATG |
| *Cox7a1* | CCGACAATGACCTCCCAGTA | ACTTCTTGTGGGGGAAGGAG |
| *Cpt1a* | GCTGCACTCCTGGAAGAAGA | GGTGTCTAGGGTCCGATTGA |
| *Cpt2* | CGGCCCTTAAGTGCTGTCT | TGGCTGTCATTCAAGAGAGG |
| *Eva1* (or *Mpzl2*) | TGTGCTTCCACTTCTCCTGA | AGTTAGCGCATCTCCCACAG |
| *Gapdh* | TtgtgatgggtgtgaaccacgA | TCTTCTGGGTGGCAGTGATGG |
| *Hspb7* | TCACCACCTTCAACAACCAC | GTGAGGCTACCATCCTCTCG |
| *Lep* | CAAGACCATTGTCACCAGGA | TCATTGGCTATCTGCAGCAC |
| *Pparg* | CAGGCCTCATGAAGAACCTT | GGATCCGGCAGTTAAGATCA |
| *Ppargc1a* | TTGCCCAGATCTTCCTGAAC | TCTGTGAGAACCGCTAGCAA |
| *Prdm16* | GAGAAGTTCTGCGTGGATGC | AGGCACCTTCTTTCACATGC |
| *Tbx1* | TGTGGGACGAGTTCAATCAG | TGTCATCTACGGGCACAAAG |
| *Tmem26* | CTTTCTCCGGCCATCTTTGT | TGGGATGACAGGGTTTGATT |
| *Ucp1* | GGCAAAAACAGAAGGATTGC | TAAGCCGGCTGAGATCTTGT |

Abbreviations:

*Adipoq*: adiponectin

*Cd137* (or *Tnfrsf9*): tumor necrosis factor receptor superfamily, member 9

*Cidea*: cell death-inducing DNA fragmentation factor, alpha subunit-like effector A

*Cox7a1*: cytochrome c oxidase subunit VIIa 1

*Cpt1a*: carnitine palmitoyltransferase 1a, liver

*Cpt2*: carnitine palmitoyltransferase 2

*Eva1* (or *Mpzl2*): myelin protein zero-like 2

*Gapdh*: glyceraldehyde-3-phosphate dehydrogenase

*Hspb7*: heat shock protein family, member 7

*Lep*: leptin

*Pparg*: peroxisome proliferator activated receptor gamma

*Ppargc1a*: peroxisome proliferative activated receptor, gamma, coactivator 1 alpha

*Prdm16*: PR domain containing 16

*Tbx1*: T-box 1

*Tmem26*: transmembrane protein 26

*Ucp1*: uncoupling protein 1
