# Supplementary material for: GNB3 overexpression causes obesity and metabolic syndrome
Source: PLoS One. 2017 Dec 5;12(12):e0188763. doi: 10.1371/journal.pone.0188763 (PMC5716578; doi:10.1371/journal.pone.0188763)
Supplement: S2 Table — (DOCX) [file pone.0188763.s008.docx]

**S2 Table. Absolute weights (g) of dissected adipose tissues and liver.**

| **Tissue** | **Age** | **Sex** | **Genotype** | **Average weight** | **SD** | **P** | **Significant?** |
| --- | --- | --- | --- | --- | --- | --- | --- |
| gWAT | 5 weeks | F | WT | 0.2927 | 0.1430 | 0.9727 | no |
|  |  |  | *GNB3*-T/+ | 0.2947 | 0.08817 |  |  |
|  |  | M | WT | 0.4255 | 0.1442 | 0.8238 | no |
|  |  |  | *GNB3*-T/+ | 0.4384 | 0.1235 |  |  |
|  | 20 weeks | F | WT | 0.5069 | 0.3776 | <0.0001 | **** |
|  |  |  | *GNB3*-T/+ | 1.297 | 0.6515 |  |  |
|  |  | M | WT | 0.7560 | 0.3061 | <0.0001 | **** |
|  |  |  | *GNB3*-T/+ | 1.357 | 0.4051 |  |  |
| iWAT | 5 weeks | F | WT | 0.3414 | 0.04497 | n/a | n/a |
|  |  |  | *GNB3*-T/+ | 0.3439 | 0.0 |  |  |
|  |  | M | WT | 0.3624 | 0.05929 | 0.5387 | no |
|  |  |  | *GNB3*-T/+ | 0.3463 | 0.03430 |  |  |
|  | 20 weeks | F | WT | 0.2465 | 0.08008 | 0.0129 | * |
|  |  |  | *GNB3*-T/+ | 0.5109 | 0.2623 |  |  |
|  |  | M | WT | 0.4532 | 0.2509 | 0.1828 | no |
|  |  |  | *GNB3*-T/+ | 0.5882 | 0.1645 |  |  |
| BAT | 5 weeks | F | WT | 0.0799 | 0.008725 | 0.9365 | no |
|  |  |  | *GNB3*-T/+ | 0.07954 | 0.01043 |  |  |
|  |  | M | WT | 0.1091 | 0.02022 | 0.9029 | no |
|  |  |  | *GNB3*-T/+ | 0.1100 | 0.01396 |  |  |
|  | 20 weeks | F | WT | 0.08075 | 0.01669 | 0.0015 | ** |
|  |  |  | *GNB3*-T/+ | 0.1135 | 0.03946 |  |  |
|  |  | M | WT | 0.1256 | 0.02986 | 0.0010 | *** |
|  |  |  | *GNB3*-T/+ | 0.1719 | 0.05021 |  |  |
| Liver | 5 weeks | F | WT | 0.9623 | 0.06720 | 0.1311 | no |
|  |  |  | *GNB3*-T/+ | 1.035 | 0.1266 |  |  |
|  |  | M | WT | 1.415 | 0.1492 | 0.2613 | no |
|  |  |  | *GNB3*-T/+ | 1.487 | 0.1427 |  |  |
|  | 20 weeks | F | WT | 1.229 | 0.1623 | 0.0169 | * |
|  |  |  | *GNB3*-T/+ | 1.385 | 0.2046 |  |  |
|  |  | M | WT | 1.423 | 0.2294 | 0.0157 | * |
|  |  |  | *GNB3*-T/+ | 1.633 | 0.1983 |  |  |

**P* < 0.05, ***P* < 0.01, ****P* < 0.001, *****P* < 0.0001 vs. WT of same sex by unpaired Student’s *t*-test.
