# Supplementary material for: GNB3 overexpression causes obesity and metabolic syndrome
Source: PLoS One. 2017 Dec 5;12(12):e0188763. doi: 10.1371/journal.pone.0188763 (PMC5716578; doi:10.1371/journal.pone.0188763)
Supplement: S3 Table — GNB3-T/+ mice and WT littermates were subjected to behavioral tests in order to evaluate anxiety/depressive-like behaviors and learning and memory. (DOCX) [file pone.0188763.s009.docx]

**S3 Table. Summary of behavioral assessment of *GNB3*-T/+ mice.** *GNB3*-T/+ mice and WT littermates were subjected to behavioral tests in order to evaluate anxiety/depressive-like behaviors and learning and memory.

| **Category** | **Test** | **# of mice** | **Measurement** | **Genotype** | **Average** | **SD** | **P** |
| --- | --- | --- | --- | --- | --- | --- | --- |
| **Mouse weight** | Mouse weight | 7 | Weight (g) | WT | 35.30 | 2.216 | 0.7618 |
|  |  | 6 |  | *GNB3*-T/+ | 34.77 | 3.880 |  |
| **Anxiety/**  **depression-like** | Novelty suppressed feeding test | 7 | Amount of sucrose eaten (mg) | WT | 216.6 | 266.5 | 0.6432 |
|  |  | 6 |  | *GNB3*-T/+ | 152.0 | 214.1 |  |
|  |  | 7 | Latency to approach (s) | WT | 63.08 | 36.37 | 0.2897 |
|  |  | 6 |  | *GNB3*-T/+ | 93.58 | 61.30 |  |
|  |  | 7 | Latency to feed (s) | WT | 468.9 | 425.4 | 0.6554 |
|  |  | 6 |  | *GNB3*-T/+ | 381.2 | 206.2 |  |
|  |  | 7 | Total time spent feeding (s) | WT | 68.70 | 69.79 | 0.5873 |
|  |  | 6 |  | *GNB3*-T/+ | 93.33 | 89.12 |  |
|  |  | 7 | Number of approaches | WT | 31.86 | 10.19 | 0.4953 |
|  |  | 6 |  | *GNB3*-T/+ | 28.00 | 9.381 |  |
|  | Marble burying | 7 | # of marbles 50% buried | WT | 7.357 | 5.352 | 0.0751 |
|  |  | 6 |  | *GNB3*-T/+ | 2.750 | 2.132 |  |
|  |  | 7 | # of marbles 67% buried | WT | 5.000 | 4.082 | 0.0554 |
|  |  | 6 |  | *GNB3*-T/+ | 1.250 | 1.332 |  |
|  | Social interaction | 6 | Latency to interact (s) | WT | 10.78 | 8.767 | 0.7800 |
|  |  | 6 |  | *GNB3*-T/+ | 12.20 | 7.328 |  |
|  |  | 6 | Total time interacting (s) | WT | 210.4 | 47.88 | 0.4568 |
|  |  | 6 |  | *GNB3*-T/+ | 185.3 | 63.38 |  |
|  | Open field | 7 | Average velocity (cm/s^2^) | WT | 6.160 | 0.7572 | 0.4014 |
|  |  | 6 |  | *GNB3*-T/+ | 6.538 | 0.8046 |  |
|  |  | 7 | Time spent in border (s) | WT | 463.3 | 188.7 | 0.6886 |
|  |  | 6 |  | *GNB3*-T/+ | 501.8 | 136.7 |  |
| **Learning/**  **memory** | Novel object recognition (Day 1 - habituation) | 7 | # of touches to object 1 | WT | 15.29 | 10.77 | 0.3650 |
|  |  | 6 |  | *GNB3*-T/+ | 21.50 | 12.97 |  |
|  |  | 7 | # of touches to object 2 | WT | 18.43 | 8.979 | 0.2472 |
|  |  | 6 |  | *GNB3*-T/+ | 28.17 | 18.83 |  |
|  |  | 7 | Time spent sniffing object 1 (s) | WT | 10.24 | 7.053 | 0.2528 |
|  |  | 6 |  | *GNB3*-T/+ | 14.41 | 4.605 |  |
|  |  | 7 | Time spent sniffing object 2 (s) | WT | 11.97 | 8.071 | 0.0966 |
|  |  | 6 |  | *GNB3*-T/+ | 30.77 | 26.15 |  |
|  |  | 7 | Latency to object 1 (s) | WT | 111.1 | 56.22 | 0.1833 |
|  |  | 6 |  | *GNB3*-T/+ | 63.16 | 65.74 |  |
|  |  | 7 | Latency to object 2 (s) | WT | 101.3 | 118.7 | 0.3579 |
|  |  | 6 |  | *GNB3*-T/+ | 180.7 | 178.2 |  |
|  |  | 7 | Distance moved (cm) | WT | 3051 | 528.8 | 0.2672 |
|  |  | 6 |  | *GNB3*-T/+ | 2672 | 641.0 |  |
|  |  | 7 | Average velocity (cm/s^2^) | WT | 5.259 | 0.7228 | 0.2065 |
|  |  | 6 |  | *GNB3*-T/+ | 4.621 | 0.9877 |  |
|  | Novel object recognition (Day 2 - habituation) | 7 | # of touches to object 1 | WT | 11.14 | 5.336 | 0.5111 |
|  |  | 6 |  | *GNB3*-T/+ | 13.83 | 8.796 |  |
|  |  | 7 | # of touches to object 2 | WT | 10.00 | 6.325 | 0.2507 |
|  |  | 6 |  | *GNB3*-T/+ | 16.00 | 11.22 |  |
|  |  | 7 | Time spent sniffing object 1 (s) | WT | 8.217 | 4.724 | 0.9248 |
|  |  | 6 |  | *GNB3*-T/+ | 8.573 | 8.364 |  |
|  |  | 7 | Time spent sniffing object 2 (s) | WT | 5.691 | 4.870 | 0.1664 |
|  |  | 6 |  | *GNB3*-T/+ | 10.52 | 6.853 |  |
|  |  | 7 | Latency to object 1 (s) | WT | 173.7 | 164.6 | 0.8476 |
|  |  | 6 |  | *GNB3*-T/+ | 151.9 | 181.0 |  |
|  |  | 7 | Latency to object 2 (s) | WT | 170.5 | 160.9 | 0.6543 |
|  |  | 6 |  | *GNB3*-T/+ | 135.0 | 105.5 |  |
|  |  | 7 | Distance moved (cm) | WT | 3175 | 499.2 | 0.0581 |
|  |  | 6 |  | *GNB3*-T/+ | 2409 | 796.3 |  |
|  |  | 7 | Average velocity (cm/s^2^) | WT | 5.557 | 0.6866 | 0.0435* |
|  |  | 6 |  | *GNB3*-T/+ | 4.261 | 1.315 |  |
|  | Novel object recognition (No delay) | 7 | # of touches to familiar object | WT | 9.429 | 4.467 | 0.2175 |
|  |  | 6 |  | *GNB3*-T/+ | 13.67 | 7.118 |  |
|  |  | 7 | # of touches to novel object | WT | 25.29 | 7.158 | 0.0363* |
|  |  | 6 |  | *GNB3*-T/+ | 16.67 | 5.610 |  |
|  |  | 7 | Time spent sniffing familiar object (s) | WT | 7.634 | 5.573 | 0.5832 |
|  |  | 6 |  | *GNB3*-T/+ | 9.547 | 6.637 |  |
|  |  | 7 | Time spent sniffing novel object (s) | WT | 22.22 | 12.10 | 0.2283 |
|  |  | 6 |  | *GNB3*-T/+ | 14.91 | 7.594 |  |
|  |  | 7 | Latency to familiar object (s) | WT | 182.1 | 159.0 | 0.5981 |
|  |  | 6 |  | *GNB3*-T/+ | 144.6 | 59.29 |  |
|  |  | 7 | Latency to novel object (s) | WT | 92.88 | 112.0 | 0.6363 |
|  |  | 6 |  | *GNB3*-T/+ | 121.3 | 96.29 |  |
|  |  | 7 | Distance moved (cm) | WT | 2594 | 842.3 | 0.2566 |
|  |  | 6 |  | *GNB3*-T/+ | 2074 | 700.1 |  |
|  |  | 7 | Average velocity (cm/s^2^) | WT | 4.479 | 1.409 | 0.2547 |
|  |  | 6 |  | *GNB3*-T/+ | 3.629 | 1.086 |  |
|  | Novel object recognition (1 hour delay) | 7 | # of touches to familiar object | WT | 12.43 | 9.693 | 0.7744 |
|  |  | 6 |  | *GNB3*-T/+ | 14.17 | 11.67 |  |
|  |  | 7 | # of touches to novel object | WT | 13.43 | 6.503 | 0.8553 |
|  |  | 6 |  | *GNB3*-T/+ | 14.50 | 13.55 |  |
|  |  | 7 | Time spent sniffing familiar object (s) | WT | 11.45 | 13.05 | 0.6128 |
|  |  | 6 |  | *GNB3*-T/+ | 8.453 | 5.574 |  |
|  |  | 7 | Time spent sniffing novel object (s) | WT | 15.54 | 15.78 | 0.7894 |
|  |  | 6 |  | *GNB3*-T/+ | 13.39 | 11.93 |  |
|  |  | 7 | Latency to familiar object (s) | WT | 121.2 | 74.14 | 0.9892 |
|  |  | 6 |  | *GNB3*-T/+ | 121.7 | 53.25 |  |
|  |  | 7 | Latency to novel object (s) | WT | 131.7 | 107.5 | 0.9755 |
|  |  | 6 |  | *GNB3*-T/+ | 129.9 | 94.47 |  |
|  |  | 7 | Distance moved (cm) | WT | 2600 | 469.6 | 0.6772 |
|  |  | 6 |  | *GNB3*-T/+ | 2455 | 745.5 |  |
|  |  | 7 | Average velocity (cm/s^2^) | WT | 4.518 | 0.8279 | 0.9788 |
|  |  | 6 |  | *GNB3*-T/+ | 4.504 | 1.036 |  |
|  | Novel object recognition (24 hour delay) | 7 | # of touches to familiar object | WT | 15.86 | 12.08 | 0.7294 |
|  |  | 6 |  | *GNB3*-T/+ | 18.17 | 11.23 |  |
|  |  | 7 | # of touches to novel object | WT | 29.43 | 17.00 | 0.6972 |
|  |  | 6 |  | *GNB3*-T/+ | 26.00 | 13.30 |  |
|  |  | 7 | Time spent sniffing familiar object (s) | WT | 10.25 | 9.741 | 0.9276 |
|  |  | 6 |  | *GNB3*-T/+ | 10.69 | 6.834 |  |
|  |  | 7 | Time spent sniffing novel object (s) | WT | 36.86 | 25.01 | 0.3557 |
|  |  | 6 |  | *GNB3*-T/+ | 23.37 | 25.30 |  |
|  |  | 7 | Latency to familiar object (s) | WT | 128.4 | 77.07 | 0.6903 |
|  |  | 6 |  | *GNB3*-T/+ | 108.9 | 95.05 |  |
|  |  | 7 | Latency to novel object (s) | WT | 71.85 | 84.80 | 0.8849 |
|  |  | 6 |  | *GNB3*-T/+ | 66.03 | 48.64 |  |
|  |  | 7 | Distance moved (cm) | WT | 3089 | 853.1 | 0.4710 |
|  |  | 6 |  | *GNB3*-T/+ | 2772 | 637.0 |  |
|  |  | 7 | Average velocity (cm/s^2^) | WT | 5.352 | 1.385 | 0.4848 |
|  |  | 6 |  | *GNB3*-T/+ | 4.848 | 1.075 |  |
